# Creativity as a Stepping Stone toward a Brighter Future

**DOI:** 10.3390/jintelligence6020021

**Published:** 2018-03-26

**Authors:** James Kaufman

**Affiliations:** Neag School of Education, University of Connecticut, 2131 Hillside Road, Unit 3007, Storrs, CT 06269-3007, USA; james.kaufman@uconn.edu

**Keywords:** creativity, IQ, meaning, fairness, ethnicity

## Abstract

If IQs continue to rise over generation, why has the world been unable to solve basic recurrent problems? This paper argues that creativity, which is overlooked in IQ tests and showing no signs of a similar increase, may be part of the reason of why the Flynn Effect has not led to a better world. Creativity’s predictive power for traditional positive outcomes, such as school or work performance, is significant but slight. However, there are other ways that creativity can help to make a better world. Two exemplar ways that are discussed in this paper are how creativity can (a) help people lead happier and more meaningful lives and (b) focus a spotlight on talented members of underrepresented groups who are overlooked by traditional measures. Both of these directions can lead to a world that is better equipped to solve larger issues.

## 1. Introduction

It is easy to glamorize the “good old days”. Many times in the past appeal to people. Some are nostalgic for eras they lived through themselves, whereas others may wonder about life in ancient Rome. However, the past held quite a few downsides. Most past generations lived in a world that was unpleasant and dangerous for people who were not wealthy men. It is also easy to forget the comforts of air conditioning, penicillin, basic safety regulations, and prenatal care (e.g., [1]). That said, there is nonetheless a feeling among many people that the world is not moving in the right direction. Although the current state of the world may still represent an improvement over past horrors, it nonetheless seems worse than a multitude of possible worlds that might exist under the same conditions. If our current global situation were a Choose Your Own Adventure book, then there is a distinct feeling that a wrong decision was made several pages back.

As this special issue asks, why are we not living in the best of times? The Flynn Effect [2,3] has consistently shown that IQ is increasing at approximately three points per decade. We are, presumably, getting smarter over time. Yet even if we are not living in the imagined future of vacationing on Venus, should not we have solved some basic problems by now? Why do hunger, lack of proper medical care, and homelessness remain predominant issues after so many years? The “duck and cover” school routine (in preparation for potential nuclear war) is gone, but it has been replaced by the equally terrifying “lockdown” routine (in preparation for a school shooter).

The obvious answer is that there’s more to life than IQ, even if our society’s priorities do not reflect this concept. The idea that we value the concepts we are able to measure dates back more than 100 years to Lord Kelvin (e.g., [4]). IQ tests have a long and varied history, and over the last 150 years there have many bursts of development and inspiration, from Wechsler’s combination of verbal and non-verbal assessments into one battery to the push in the 1980’s to develop theory-based IQ tests [5]. Yet, innovations are still the exception, and there has been little advancement in the last decade or two. Technological advances have enabled a new world of testing possibilities (e.g., [6]), yet IQ tests have been content to remain stagnant. Increasing IQs may be notable, but they may be less related to our ability as a population to cope with ever-changing problems and possibilities.

What do IQ tests miss? In the pages that follow, I will explore why creativity’s absence from IQ tests (and, therefore, any Flynn effect boost) is a key reason that the world is not on a strong forward trajectory. It is important to note before I discuss creativity that just as IQ alone is not enough to solve global problems, neither is creativity. They are but two of many constructs (many discussed in this issue, such as wisdom or rational thought) that are needed to create the leaders and humanitarians of tomorrow [7]. I am under no myopic illusion that all problems would be solved if everyone magically woke up tomorrow being twice as creative as they are today. Indeed, creativity is not always benevolent [8]. J. Robert Oppenheimer was an incredibly creative (and smart) scientist who oversaw the Manhattan Project [9]. It is possible to argue that nuclear weapons have done much good for the world (i.e., avoiding potential world wars), but it is impossible to argue that nuclear weapons have not unleashed destruction and death. Positive creative actions can be used to prevent negative creative actions, such as current anti-terrorism units [10]. However, the potential misuse of creativity cannot be ignored. Although I will focus this article on the positive potential of creativity, I am quite aware that creativity can (and has) been used to make the world worse, as well.

Another important note, that whereas IQ may be increasing, creativity is probably not. In a well-publicized study, Kim [11] analyzed archival data on the figural form (i.e., drawing) of the Torrance Tests of Creative Thinking from six normative samples between 1966 and 2008. She found that as of 1990, scores began to decline. Unfortunately, scores from the verbal form were not analyzed. Creativity is a complex construct that manifests itself differently depending the domain [12,13]. A subsequent study analyzed actual creative writing and visual art that were published between 1990 and 2011 [14]. In contrast to Kim’s [11] study, many components of visual art (such as complexity and unconventionality) showed an increase over the two decades. However, the creative writing was rated as more formulaic and conventional. Without significantly more research, it is hard to reach any conclusion about trends, but there is little evidence that any Flynn-like effect is occurring in creativity.

When creativity is studied, the vast majority of researchers (more than 70%) focus on how to predict or increase creativity [15]. This work is important, but it leaves a gap for someone trying to argue creativity’s importance in potentially solving world problems. There are many papers describing the personality, motivational, cognitive, or situational traits that can increase (or decrease) creativity. Yet, few consider how creativity can predict positive outcomes. One initial suggestion is to increase this type of work.

When this predictive research is conducted, however, the desirable outcomes that were studied tend to be standard, such as academic achievement [16], job performance [17], or employee satisfaction [18]. Although creativity is positively related to these variables, school and work success are better predicted by intelligence and conscientiousness [19,20].

If we want to talk about how creativity can change the world, we need to consider new pathways. An argument that focuses on easily-measured school or work performance can be quickly countered: why go through the hassle of testing creativity when there are more established assessments of intelligence (and personality) that are better predictors? Further, there is much more to life than GPA or a supervisor’s rating. I will discuss ways in which creativity can help to solve the world’s problems at all levels—from personal to global [21]. With further empirical studies to add to our base of knowledge, creativity may be better used to help people.

## 2. Changing the World in New Ways

It is instinctual to think of large-scale, genius-level interventions when we think about changing the world, such as the development of the internet or the Polio vaccine. Certainly, with world leaders and political parties in seemingly perpetual states of disagreement and inability to work together, a successful mediator would need creative solutions. In addition, creativity is a driving force behind most of the advances we have made [22]. But greatness does not suddenly materialize. Child prodigies, for example, are only barely more like to develop into adult geniuses than anyone else [23]. Although we know a great deal about which variables predict Big-C, or creative geniuses [24]. Millions of people may have the same perfect blend of familial and cultural environments and ideal individual factors yet accomplish nothing meaningful. A top-down approach may not be the best way to identify how creativity can help to change the world.

One place to start is that there is no Big-C without mini-c (personal creativity), little-c (everyday creativity), or Pro-c (expert-level creativity; [25,26]). If creativity is not nurtured in school at the K-12 [27] and collegiate [28] level, then it will be much less prevalent in adults. In addition, creativity has a multitude of benefits beyond low-level correlations with school and work performance. For example, in our daily lives, creativity is associated with being seen as more sexually desirable [29], better physical health [30], and higher career satisfaction [31].

I will now address two understudied, bottom-up ways in which creativity can lead to a better world: through providing and maintaining meaning in life and by increasing equity and fairness.

### 2.1. Meaning and Well Being 

Creativity has long been a key part of humanistic (e.g., [32,33]) and positive psychology [34,35]. Creative activity can not only help people experience personal growth and be more likely to contribute to the world; it can also help to prevent them from focusing on the looming certainty that everyone is eventually going to die [36]. Lifton [37] suggests there are many ways that people can accept their own mortality by focusing on what may keep their memory or presence alive after death. In addition to such possibilities as family or spiritual beliefs, one way is through creative activity. As Kaufman [38] suggests, there are many ways that creativity can help people find meaning in life. Writing narratives or memoirs, creating art, or finding a unique creative passion may help someone make sense of their own life [39], experience joy in the present moment [40], manage existential worries about death [41], and leave a legacy for future generations [42].

In addition to enhancing someone’s meaning in life on a larger scale, creativity can help hold on to meaning after experiencing a traumatic event. Being creative is associated with people who continue to thrive and grow after experiencing a traumatic event [43]. Although culture and individual differences play an important role, these general connection has been found with survivors of such large-scale disasters as the Rwandan massacre [44] or Hurricane Katrina [45]. On a smaller scale, creativity can help reduce stress [46], lift psychological burdens [47], and replenish a person after a difficult day at work [48]. The exact underlying nature of creativity’s contribution to these positive outcomes does need more study [49].

It is reasonable to question how creativity leading to personal meaning and well-being can impact the world. It turns out that happy people are much more likely to engage in behaviors that are beneficial to other people. For example, general well-being is associated with being more likely to participate in general prosocial activities [50]. Happier people are specifically more likely to help the environment [51], donate to charities [52], and be more productive at work [53]. They are also less likely to engage in criminal or delinquent activity [54]. The impact of general well-being on society is strong enough that Maccagnan, Wren-Lewis, Brown, and Taylor [55] suggest that it may be just as important to accurate assess and nurture the happiness of a society as it is to consider more traditional measures of a country’s success, such as its GDP (gross domestic product).

### 2.2. Equity and Fairness

Most measures of ability and achievement (IQ tests, SATs, GREs, and many others) show significant differences by ethnicity [56,57], with White and Asian Americans tending to receive higher scores than other groups. Achievement tests show differences by gender, with women receiving lower scores on math-related measures [58]. This topic is both controversial and nuanced, and as such beyond the scope of the current paper. Many questions of potential bias have been raised, and I have discussed this issue in more detail elsewhere [59,60].

As I have noted before [61,62], measures of creativity either show virtually no differences by ethnicity [63,64,65] or a slight advantage for underrepresented groups [66,67,68,69]. Gender differences are inconsistent and are generally rare [70]. On the few occasions that creativity assessments have been used to supplement (not replace) more traditional measure for college admissions, underrepresented groups have been more likely to be admitted to college—and the average SAT scores of accepted students actually increased [71]. Indeed, students accepted to Tufts who completed the supplemental measures (including creativity) were more likely to get better grades than those who did not (controlling for high school GPA and SAT scores); they were also more likely to get involved in leadership or extracurricular activities [72].

There are many obstacles to using creativity as a high-stakes measure, whether for college [61] or gifted [73] admissions. Current creativity assessments tend be either outdated or require extensive financial or personnel resources (e.g., [74]). Consider, however, the strong possibility of discovering exciting new candidates who might score lower on traditional measures. The chance to attend more prestigious colleges will offer members of underrepresented groups better access to more important and higher status jobs. Substantial research indicates that groups that are comprised of different ethnicities are more creative, effective, and produce higher quality work [75,76,77]. If we want to have better large-scale ideas in our organizations, we need to start recognizing a broader array of talents much earlier. As Wordsworth said, “the child is the father of the man” ([78], p. 7).

Another intriguing possibility is the link between getting people to counter stereotypes and an increase in creativity [79,80,81]. Although most studies are more focused on how these interventions can increase creativity, it has been proposed that the reverse angle may be equally true. In other words, helping people to increase their creativity may lead to the rejection of stereotypical thinking [82,83].

## 3. Concluding Thoughts

Is creativity an automatic pathway toward solving our world’s many problems? Of course not. But, if we move beyond the links between creativity and school and workplace performance, some intriguing possibilities emerge. Existing studies suggest that higher levels of creativity may enable people to have more meaning in their life and to be happier. Including creativity tests as part of gifted or college admissions may recognize talented members of underrepresented groups. Interventions to increase creativity may increase tolerance. Much more research is needed to establish the best ways in which creativity can enhance fairness and meaning, as well as identifying new positive outcomes that can be nurtured by creativity.

These steps will not suddenly end world hunger, climate change, or growing hatred between cultural, ethnic, and political groups. Investing in creativity is rarely a short-term solution. But, over time, people who are happier, more engaged with life, interacting with diverse groups, and more tolerant of others will be the ones who can bring us closer to the world that many of us might imagine only as a fantasy. Creativity represents a solid starting point for the future.
